# Religion and Aging in a Context of Secularization

**DOI:** 10.1093/geroni/igaa057.2267

**Published:** 2020-12-16

**Authors:** Ellen Idler

**Affiliations:** Emory University, Atlanta, Georgia, United States

## Abstract

Religion and aging has been a persistent topic of interest to gerontologists, notably Vern Bengtson over his long career. It is increasingly obvious that this research has taken place against a decades-long backdrop of declining religious attendance, with each successive cohort showing lower levels of participation. Data come from the Health and Retirement Study, a representative sample of the noninstitutionalized US population (N=20,091), ages 24-107. We examined the patterns of religious involvement during the period 2004-2014 stratified by five age groups, 24-49, 50-64, 65-74, 75-89, 90+. Attending religious services has an age-graded pattern; each older cohort has a higher level of religiosity than the one following it, with the exception of those 90. Patterns for other measures of religious involvement are less dramatic but similar in direction. Lower levels of religious participation in younger cohorts imply a smaller proportion for whom these protective social ties are available. Part of a symposium sponsored by the Religion, Spirituality and Aging Interest Group.

